# Monte Carlo calculation of beam quality correction for solid‐state detectors and phantom scatter correction at ^137^Cs energy

**DOI:** 10.1120/jacmp.v15i1.4445

**Published:** 2014-01-06

**Authors:** T. Palani Selvam, Subhalaxmi Mishra, R.S. Vishwakarma

**Affiliations:** ^1^ Radiological Physics and Advisory Division, Health, Safety and Environment Group Bhabha Atomic Research Centre Mumbai 400 085 India

**Keywords:** Monte Carlo, brachytherapy, energy response, phantom scatter

## Abstract

Beam quality correction $k_{QQ_{0}}\;(r)$, which reflects the absorbed energy dependence of the detector, is calculated for solid‐state detector materials diamond, LiF, ${\text{Li}}_{2}B_{4}O_{7}$, and ${\text{Al}}_{2}O_{3}$ for the ${}^{137}\text{Cs}$ RTR brachytherapy source using the Monte Carlo‐based EGSnrc code system. The study also includes calculation of detector‐specific phantom scatter corrections $k_{phan}(r)$ for solid phantoms such as PMMA, polystyrene, RW1, solid water, virtual water, and plastic water. Above corrections are calculated as a function of distance r along the transverse axis of the source. $k_{QQ_{0}}\;(r)$ is about unity for the ${\text{Li}}_{2}B_{4}O_{7}$ detector. LiF detector shows a gradual decrease in $k_{QQ_{0}}\;(r)$ with r (decrease is about 2% over the distance range of 1‐15 cm). Diamond detector shows a gradual increase in $k_{QQ_{0}}\;(r)$ with r (about 3% larger than unity at 15 cm). In the case of ${\text{Al}}_{2}O_{3}$ detector, $k_{QQ_{0}}\;(r)$ decreases with r steeply (about 14% over the distance range of 1‐15 cm). The study shows that some solid‐state detectors demonstrate distance‐dependent $k_{phan}(r)$ values, but the degree of deviation from unity depends on the type of solid phantom and the detector.

PACS number: 87.10.Rt, 87.53.Bn, 87.53.Jw, 87.56.Bg

## I. INTRODUCTION

American Association of Physicists in Medicine (AAPM) Task Group reports AAPM TG43^(1)^ and TG43U 1^(2)^ recommend water as a reference medium for dosimetry of interstitial brachytherapy sources. Due to high‐dose gradients near brachytherapy sources and specification of the dose parameters within few centimeters of the source, source‐detector distance should be specified very accurately for dosimetric measurements. Precise positioning of detectors, reproducibility of source and detectors in reference liquid water medium, and water proofing of detectors posses a practical problem. Solid phantom materials can be easily machined to accommodate the source and detectors in a precise geometrical configuration, facilitating an accurate measurement and reproducibility in source‐detector geometry.

In a previously published article, relative absorbed‐dose energy response corrections R for detector materials such as air, LiF, ${\text{Li}}_{2}B_{4}O_{7}$, Si diode, diamond, and ${\text{Al}}_{2}O_{3}$ were presented for ${}^{169}\text{Yb}$ and ${}^{125}I$ brachytherapy sources.^(3)^ The corrections were calculated using the EGSnrc‐based^(4)^ Monte Carlo code system for liquid water, PMMA, and polystyrene phantom materials. The present study is aimed at investigating absorbed‐dose energy dependence of solid‐state detector materials such as diamond, LiF, ${\text{Li}}_{2}B_{4}O_{7}$, and ${\text{Al}}_{2}O_{3}$ at the ${}^{137}\text{Cs}$ energy. This investigation also includes calculation of detector‐specific phantom scatter correction for different solid phantoms such as PMMA, polystyrene, RW1, solid water, virtual water, and plastic water. The EGSnrc‐based^(4)^ user‐codes DOSRZnrc and FLURZnrc^(5)^ are used in the study.

## II. MATERIALS AND METHODS

### A. RTR ${}^{137}\text{Cs}$ source

The geometric details and material data of the RTR ${}^{137}\text{Cs}$ are from the published work.^(6)^ The active length and active radius (active material is gold) of the source are 1.5 cm and 0.04 cm, respectively. The outer radius of the source is 1.5 mm. For Monte Carlo calculations, we have considered only the 662 keV gamma energy of ${}^{137}\text{Cs}$ emission, as in a previously published study by Selvam et al.,^(7)^ it was demonstrated that ${}^{137}\text{Ba}$ X‐rays were not important.

### B. Phantom materials

Elemental composition, mass fraction, mass density, <Z/A>, and effective atomic number ($Z_{\text{eff}}$) of water and solid phantom materials are presented in Table 1. The atomic composition and density details of the phantoms are taken from the literature.^8^, ^9^, ^10^, ^11^ $Z_{\text{eff}}$ values are calculated at 662 keV using the ${\text{Auto}-Z}_{\text{eff}}$ software by Taylor et al.^(12)^

**Table 1:** Elemental composition, mass fraction, mass density, <Z/A>, and effective atomic number ($Z_{\text{eff}}$) of water and solid phantom materials

*Element*	*Z*	*A*	*Water* ^a^	*Solid water* ^b^	*RW1* ^b^	*Plastic Water* ^c^	*Virtual Watef* ^d^	*PMMA* ^a^	*Polystyrene* ^a^
Composition and mass fraction									
H	1	1.008	0.1119	0.081	0.132	0.0930	0.077	0.08054	0.07742
C	6	12.01		0.672	0.794	0.6282	0.687	0.59985	0.92258
N	7	14.01		0.024		0.0100	0.023		
O	8	15.99	0.8881	0.199	0.038	0.1794	0.189	0.31961	
Mg	12	24.31			0.009				
Cl	17	35.46		0.001	0.027	0.0096	0.001		
Ca	20	40.08		0.023		0.0795	0.023		
Br	35	79.90				0.0003			
Mass density (g/cm^3^)		1.000	1.036	0.970	1.013	1.030	1.190	1.060
<Z/A>			0.555	0.540	0.565	0.545	0.538	0.539	0.538
$Z_{\text{eff}}$ ^e^			3.34	3.57	2.81	3.44	3.64	3.60	3.50

a ^a^ Hubble & Seltzer (Ref. 11);

b ^b^ ICRU‐44 ( Ref. 8);

c ^c^ Meigooni et al. (Ref. 9);

d ^d^ Reniers et al. (Ref. 10);

e ^e^ Taylor et al. (Ref. 12).

### C. Theoretical background of measurement of absorbed dose to water at brachytherapy energies

#### C.1 Dose measurements in water phantom

Following discussion is based on the published study by Adolfsson et al.^(13)^ Primary standards of absolute measurements of absorbed dose to water $D_{w}$ are based on water calorimetry.^(14)^ ${}^{60}\text{Co}$ or megavoltage (MV) photon beam serves as a reference beam quality $Q_{0}$ for this purpose. A dosimeter, for example, ionization chamber calibrated to measure $D_{w}$ at the primary or secondary standards can be used in other beam quality Q (example, other clinical MV photon beams) by using the beam quality correction factor $k_{QQ_{0}}$.^15^, ^16^ The other dosimeters, such as solid‐state dosimeters, can therefore be calibrated to measure $D_{w}$ at Q traceable to the primary standard. Note that $k_{QQ_{0}}$ may be calculated at a brachytherapy beam quality, Q, involving a solid‐state detector.

Consider a solid‐state detector is used for measuring $D_{w}$ at $Q_{0}$. This quantity is denoted by $D_{w,Q_{0}}$ The output measured by the solid‐state detector is denoted by $M_{det,Q_{0}}$. An absorbed dose‐to‐water calibration coefficient $N_{D_{W},Q_{0}}$ can be obtained by using the following the relation:

(1) $$N_{D_{w},Q_{0}}=\frac{D_{w,Q_{0}}}{M_{\text{det},Q_{0}}}$$

The absorbed dose to the material of the sensitive detector element at $Q_{0}$, $D_{det,Q_{0}}$, and $M_{det,Q_{0}}$ are related as follows:^17^, ^18^, ^19^

(2) $$D_{\text{det},Q_{0}}=k_{Q_{0}}M_{\text{det},Q_{0}}$$

where the function $k_{Q_{0}}$ (called intrinsic energy‐dependence^17^, ^18^) relates $M_{det,Q_{0}}$ and $D_{det,Q_{0}}$ as below:

(3) $$k_{Q_{0}}=\frac{D_{\text{det},Q_{0}}}{M_{\text{det},Q_{0}}}$$

Let us now consider a cylindrical photon emitting brachytherapy source (beam quality Q, in this study it is ${}^{137}\text{Cs}$) is immersed in a liquid water phantom. The absorbed dose to water in the liquid water phantom at r along the transverse axis of the source is denoted by $D_{w,Q}\;(r)$. The output measured by the detector at r is $M_{det,Q}\;(r)$. Likewise Eq. (3), absorbed dose to the detector at Q, $D_{det,Q}\;(r)$ and $M_{det,Q}\;(r)$ are related by

(4) $$k_{Q}(r)=\frac{D_{\text{det},Q}(r)}{M_{\text{det},Q}(r)}$$

$D_{w,Q}\;(r)$ is obtained by using the following relation:

(5) $$D_{w,Q}(r)=M_{\text{det},Q}(r)N_{D_{w}Q_{0}}k_{QQ_{0}}(r)$$

where $k_{QQ_{0}}\;(r)$ is the beam quality correction and is given by

(6) $$k_{QQ_{0}}(r)=\frac{D_{w,Q}(r)}{M_{\text{det},Q}(r)N_{D_{w}Q_{0}}}$$

Using Eq. (1) in Eq. (6) gives

(7) $$k_{QQ_{0}}(r)=\frac{D_{w,Q}(r)}{M_{\text{det},Q}(r)\frac{D_{w,Q_{0}}}{M_{\text{det},Q_{0}}}}=\frac{{\left[\frac{D_{w}(r)}{M_{\text{det}}(r)}\right]}_{Q}}{{\left[\frac{D_{w}}{M_{\text{det}}}\right]}_{Q_{0}}}$$

Using Eqs. (3) and (4) in Eq. (7) gives

(8) $$k_{QQ_{0}}(r)=\frac{k_{Q}(r)D_{w,Q}(r)}{D_{\text{det},Q}(r)\frac{D_{w,Q_{0}}}{D_{\text{det},Q_{0}}}k_{Q_{0}}}=\frac{k_{Q}(r)}{k_{Q_{0}}}\frac{\left[D_{w,Q}(r)/D_{\text{det},Q}(r)\right]}{\left[D_{w,Q_{0}}/D_{\text{det},Q_{0}}\right]}$$

(9) $$k_{QQ_{0}}(r)=f_{QQ_{0}}\frac{\left[D_{w,Q}(r)/D_{\text{det},Q}(r)\right]}{\left[D_{w,Q_{0}}/D_{\text{det},Q_{0}}\right]}$$

(10) $$k_{QQ_{0}}(r)=f_{QQ_{0}}/R_{QQ_{0}}$$

(11) $$\text{where}\;f_{QQ_{0}}=k_{Q}(r)/k_{Q_{0}}$$

(12) $$R_{QQ_{0}}=[D_{\text{det},Q}(r)/D_{w,Q}(r)]/[D_{\text{det},Q_{0}}/D_{w,Q_{0}}]$$

$R_{QQ_{0}}$ is relative absorbed dose energy response correction.^3^, ^17^, ^18^ As described in the previously published work,^3^, ^17^, ^18^, ^19^ absorbed‐dose dependence at Q, f(Q) relates absorbed dose to medium of interest (usually water), $D_{w,Q}$ and absorbed dose to detector, $D_{det,Q}$, as below:

(13) $$f(Q)=\frac{D_{w,Q}}{D_{\text{det},Q}}$$

Similarly at $Q_{0}$:

(14) $$f(Q_{0})=\frac{D_{w,Q_{0}}}{D_{\text{det},Q_{0}}}$$

Equation (12) is therefore written as:

(15) $$R_{QQ_{0}}=\left[1/f(Q)\right]/\left[1/f(Q_{0})\right]$$

Equation (10) has two components: (a) *f*_*QQ*0_, relative intrinsic energy dependence of the detector which can only be determined experimentally, and (b) $1/R_{QQ_{0}}$, inverse of relative absorbed‐dose energy response correction. Investigations on photon energy dependence of LiF:Mg, Ti TLDs were published in the 1960s and 1970s, with a summary of the results presented by Budd et al.^(20)^ Most of the studies measured an intrinsic energy dependence that was greater than unity for photon energies below about 150 keV, relative to TLDs that had been calibrated using ${}^{60}\text{Co}$ photons. On average, the measured light output was about 10% higher than would be expected based solely on the absorbed‐dose energy dependence. For detailed discussion on intrinsic energy dependence of TLDs, readers may consult the literature.^(17)^

As mentioned by Adolfsson et al.,^(13)^ when an ion chamber is used, $f_{QQ_{0}}=W/W_{0}$ where *W* is the mean energy imparted to air to form an ion pair in air at Q, and $W_{0}$ is the corresponding quantity at $Q_{0}$. The value of W is usually considered to be independent of the beam quality in MV photon and electron beams, but may take other values in beams of protons and heavier charged particles due to the increased ion density along the tracks of the heavy charged particles compared to that along electron tracks.^(21)^ Note that if the yield of radiation‐induced products in the detector is independent of the radiation beam quality (i.e., yield is constant), then $k_{Q}=k_{Q_{0}}$ Therefore Eq. (9) becomes

(16) $$k_{QQ_{0}}(r)\overset{cons\text{t y}ield}{=}\frac{\left[D_{w,Q}(r)/D_{\text{det},Q}(r)\right]}{\left[D_{w,Q_{0}}/D_{\text{det},Q_{0}}\right]}$$

#### C.2 Brachytherapy dose measurements in a solid phantom

Generally, in brachytherapy, absorbed dose measurements involving solid‐state detectors are carried out in solid phantoms. The absorbed dose to detector at r in the solid phantom at Q is denoted by $D_{det,\;phan,Q}(r)$. It is recalled that $D_{det,Q}(r)$ is absorbed dose to detector at Q at r in the liquid water phantom. $D_{det,Q}(r)$ and $D_{det,\;phan,Q}(r)$ are related as follows:

(17) $$D_{\text{det},Q}(r)=D_{\text{det},phan,Q}(r)k_{phan}(r)$$

where $k_{phan}\;(r)$ accounts for influence of solid phantom on the response of the detector, which is known as phantom scatter correction at beam quality Q. Therefore, when measurements are carried out in solid phantoms at Q, in addition to the application of $k_{QQ_{0}}(r)$ (Eq. (16)), the detector response is required to be corrected for $k_{phant}\;(r)$ to account of phantom scatter. The final expression for obtaining absorbed dose to water in the liquid water phantom is given by

(18) $$D_{w,Q}(r)=M_{\text{det},phan,Q}(r)k_{phan}(r)N_{D_{w},Q_{0}}k_{QQ_{0}}(r)$$

where $D_{det,\;phan,Q}(r)$ is output measured by the solid detector at Q in a solid phantom at r.

### D. Monte Carlo calculations

#### D.1 *FLURZnrc simulations of collision kerma and mean energies for* ${}^{137}\text{Cs}$ *RTR source*

The approach adapted for the Monte Carlo calculations of dose ratio of detector to water is as described in the published study.^(3)^ The source is positioned at the centre of a 40 cm diameter by 40 cm height cylindrical phantoms (liquid water and solid phantoms). The photon fluence spectrum in 10 keV energy intervals is scored along the transverse axis of the source (r=1−15 cm) in 2 mm high and 0.5 mm thick cylindrical shells. The fluence spectrum is converted to collision kerma to water and collision kerma to detector materials by using the mass energy‐absorption coefficients of water and detector materials, respectively. ^(11)^

#### D.2 *Calculations of dose ratios at* $Q_{0}$

In the published study,^(3)^ it was demonstrated that the detector‐to‐water dose ratio $[D_{\text{det}}/{D_{wat}]}_{Q_{0}}$ calculated at the reference beam quality $Q_{0}$ (${}^{60}\text{Co}$ beam) at 0.5 mm depth in water phantom was independent of the detector thickness (0.1 mm−5 mm). In the present study, we calculated the above dose ratio for depths 5 cm and 10 cm along the central axis of the water phantom. We used detector dimensions of 5 mm radius×1 mm thickness. In the Monte Carlo calculations, a parallel ${}^{60}\text{Co}$ beam is incident on a 20 cm radius×40 cm height cylindrical water phantom. The beam has a radius of 5.64 cm at the front face of the phantom (field size is 100 cm^2^). A realistic ${}^{60}\text{Co}$ spectrum from a telecobalt unit distributed along with the EGSnrc code system^(4)^ is used in the calculations. This investigation produced similar dose ratios as obtained at 5 mm depth. This suggests that $[D_{\text{det}}/{D_{wat}]}_{Q_{0}}$ is independent of depth in the water phantom. We also calculated the dose ratio at depths 5 mm, 5 cm, and 10 cm in the PMMA phantom using the detector dimensions 5 mm radius×1 mm thickness. The results obtained from the PMMA phantom compare well with the results of water phantom. We have therefore used the values of $[D_{\text{det}}/{D_{wat}]}_{Q_{0}}$ published in the previous work^(3)^ for deriving $k_{QQ_{0}}\;(r)$.

#### D.3 Monte Carlo parameters

Up to 2.5 × 10^9^ photon histories are simulated. The 1 g statistical uncertainties on the calculated absorbed dose and collision kerma values are about 0.2%. The statistical uncertainties on the calculated values of $k_{QQ_{0}}(r)$ and $k_{phan}\;(r)$ are less than 0.5%. The values of Monte Carlo parameters AE, AP, ECUT, PCUT, and ESAVE used in the FLURRZnrc calculations are 0.521, 0.01, 0.01, 2, and 2 MeV, respectively. In the case of DOSRZnrc calculations, the value of ECUT used is 0.521 MeV (10 keV kinetic energy of electrons) and the values of other parameters are as that used in the FLURRZnrc simulations. The parameters AE and AP are the low‐energy thresholds for the production of knock‐on electrons and secondary bremsstrahlung photons, respectively. The parameters ECUT and PCUT electron and photon transport cutoff, respectively. ESAVE is a parameter related to range rejection technique.

## III. RESULTS & DISCUSSION

### A. Fluence‐weighted mean energy, $E_{\text{fl}}$

Table 2 presents the values of $E_{\text{fl}}$ as a function of r for the ${}^{137}\text{Cs}$ RTR source in various phantoms. As r increases $E_{\text{fl}}$ decreases, but the degree of decrease depends on the type of phantom. For the phantoms such as water, virtual water, RW1, and solid water, $E_{\text{fl}}$ decreases from about 565 keV to 260 keV when the distance is increased from 1 cm to 15 cm. In the case of plastic water phantom, $E_{\text{fl}}$ decreases from 570 keV to 285 keV in the above distance range. The values of $E_{\text{fl}}$ at 15 cm are 228 keV and 239 keV, respectively, for PMMA and polystyrene phantoms.

**Table 2:** Monte Carlo‐calculated values of fluence‐weighted mean energy for different phantoms. The values are presented as a function of distance along the transverse axis of the ${}^{137}\text{Cs}$ RTR brachytherapy source

	*Fluence‐weighted mean energy (MeV)*						
*Distance, r (cm)*	*Water*	*PMMA*	*Polystyrene*	*Plastic Water*	*Virtual Water*	*RW1*	*Solid Water*
1	0.566	0.557	0.563	0.568	0.567	0.567	0.566
2	0.516	0.499	0.510	0.521	0.517	0.517	0.516
3	0.471	0.449	0.462	0.480	0.473	0.472	0.472
4	0.433	0.407	0.421	0.445	0.436	0.435	0.433
5	0.401	0.371	0.387	0.414	0.404	0.401	0.402
6	0.374	0.342	0.358	0.390	0.375	0.374	0.375
7	0.351	0.318	0.334	0.369	0.353	0.351	0.351
8	0.332	0.299	0.313	0.351	0.334	0.330	0.333
9	0.315	0.282	0.295	0.334	0.318	0.315	0.316
10	0.302	0.267	0.281	0.322	0.304	0.300	0.302
11	0.289	0.256	0.267	0.311	0.292	0.288	0.291
12	0.279	0.246	0.258	0.302	0.282	0.277	0.281
13	0.271	0.239	0.249	0.295	0.275	0.269	0.273
14	0.265	0.232	0.243	0.289	0.269	0.264	0.268
15	0.260	0.228	0.239	0.285	0.263	0.259	0.264

### B. Phantom scatter correction $k_{phan}\;(r)$

**Figure 1:**
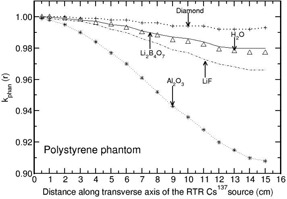
Phantom scatter correction $k_{phan}\;(r)$ presented for polystyrene phantom as a function of distance along the transverse axis of the ${}^{137}\text{Cs}$ RTR brachytherapy source. The values are presented for detector materials LiF, ${\text{Li}}_{2}B_{4}O_{7}$, diamond, ${\text{Al}}_{2}O_{3}$, and water.

**Figure 2:**
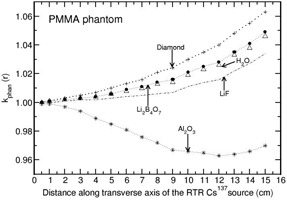
Same as Fig. 1, but for PMMA phantom.

**Figure 3:**
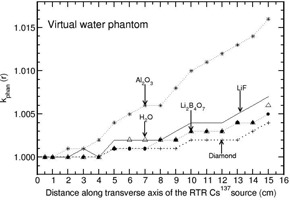
Same as Fig. 1, but for virtual water phantom.

**Figure 4:**
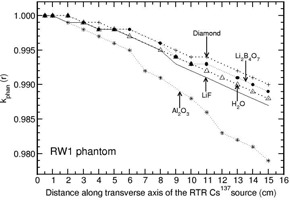
Same as Fig. 1, but for RW1 phantom.

**Figure 5:**
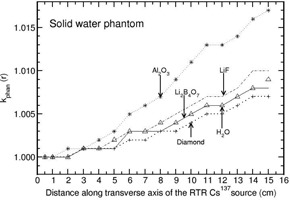
Same as Fig. 1, but for solid water phantom.

**Figure 6:**
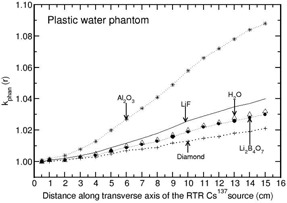
Same as Fig. 1, but for plastic water phantom.

The investigation of phantom scatter also included water as a detector material. Values of $k_{phan}(r)$ calculated for the phantoms polystyrene, PMMA, virtual water, RW1, solid water, and plastic water are presented in Figs. 1 to 6. A solid phantom may be termed as water‐equivalent when the value of $k_{phan}(r)$ is unity. The investigation suggests that some solid‐state detectors demonstrate distance‐dependent $k_{phan}\;(r)$ values, but the degree of dependence depends on the type of solid phantom and the type of detector. For example, the phantoms such as RW1, virtual water, and solid water almost behave like water‐equivalent at all distances (1‐15 cm) for all the investigated detectors (with a maximum deviation of about 2% from unity for the ${\text{Al}}_{2}O_{3}$ detector in RW1 phantom). Polystyrene, virtual water, RW1, and solid water phantoms are water‐equivalent for the diamond detector as $k_{phan}\;(r)$ is about unity, independent of distance (maximum deviation is about 1% in the distance range of 1‐15 cm for polystyrene phantom). Whereas, for the phantoms PMMA and plastic water, $k_{phan}(r)$ increases with r for the diamond detector. The value increases to 1.0607 in PMMA and 1.0212 in plastic water at 15 cm for the diamond detector. For the LiF, ${\text{Li}}_{2}B_{4}O_{7}$ detectors, virtual water, RW1, and solid water are water‐equivalent (within 1%). Note that ${\text{Li}}_{2}B_{4}O_{7}$ detector behaves like water detector at all distances for all the solid phantom materials investigated. For the ${\text{Al}}_{2}O_{3}$ detector, the phantoms such as Polystyrene, PMMA, and RW1 show decrease in $k_{phan}(r)$ with r and the degree of decrease is higher for polystyrene phantom. For example, the value decreases to 0.9075, 0.9697, and 0.9794 at 15 cm for the phantoms polystyrene, PMMA, and RW1, respectively. The degree of decrease is higher in polystyrene phantom.

### C. Beam quality correction $k_{QQ_{0}}\;(r)$

**Figure 7:**
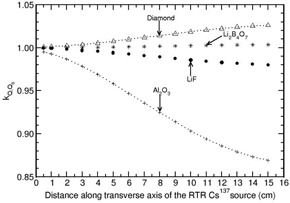
Monte Carlo‐calculated beam quality correction $k_{QQ_{0}}\;(r)$ for ${}^{137}\text{Cs}$ RTR brachytherapy source. The values are presented as a function of distance along the transverse axis of the source for detector materials LiF, ${\text{Li}}_{2}B_{4}O_{7}$, diamond, and ${\text{Al}}_{2}O_{3}$.

Figure 7 presents the values of $k_{QQ_{0}}\;(r)$ for the ${}^{137}\text{Cs}$ RTR source obtained using Eq. (16). The numerical values of this figure are given in Table 3. For the ${\text{Li}}_{2}B_{4}O_{7}$ detector, $k_{QQ_{0}}\;(r)$ is about unity, and is independent of r. The LiF detector shows a gradual decrease in $k_{QQ_{0}}\;(r)$ with r. The decrease is 2% over the distance range of 1‐15 cm. Diamond detector shows a gradual increase in $k_{QQ_{0}}\;(r)$ with r (about 3% larger than unity at 15 cm). For the ${\text{Al}}_{2}O_{3}$ detector, $k_{QQ_{0}}\;(r)$ decreases with r steeply (about 14% over the distance range of 1–15 cm).

**Table 3:** Monte Carlo‐calculated values of beam quality correction $k_{QQ_{0}}\;(r)$. The data are presented as a function of distance along the transverse axis of the ${}^{137}\text{Cs}$ RTR source

	$k_{QQ_{0}}\;(r)$			
*Distance, r (cm)*	*Diamond*	${\text{Al}}_{2}O_{3}$	${\text{Li}}_{2}B_{4}O_{7}$	*LiF*
1	1.001	0.993	1.000	0.999
2	1.002	0.987	1.000	0.998
3	1.004	0.978	1.000	0.997
4	1.005	0.969	1.000	0.996
5	1.007	0.958	1.001	0.994
6	1.010	0.947	1.001	0.993
7	1.012	0.936	1.001	0.991
8	1.014	0.925	1.002	0.989
9	1.016	0.914	1.002	0.987
10	1.018	0.903	1.002	0.986
11	1.021	0.894	1.003	0.984
12	1.022	0.886	1.003	0.983
13	1.024	0.879	1.003	0.982
14	1.025	0.873	1.004	0.981
15	1.026	0.869	1.004	0.980

### D. Influence of detector dimensions on detector response

Dimensions of TLD‐100 (LiF:Mg,Ti) chips reported in the literature^22^, ^23^ are $3\times 3\times 0.9\;{\text{mm}}^{3}$, and $1\times 1\times 1\;{\text{mm}}^{3}$, and $3.2\times 3.2\times 0.38\;{\text{mm}}^{3}$. Carbon‐doped cylindrical discs of ${\text{Al}}_{2}O_{3}$ detectors (4 mm diameter×1 mm height) are used in radiotherapy photon beams.^(24)^ ${\text{Al}}_{2}O_{3}$: C chips (2 mm long and $0.5\times 0.5\;{\text{mm}}^{2}$ in cross‐sectional area) are used in ^192^Ir high‐dose‐rate dosimetry.^(25)^ The sensitive volume of the PTW/diamond detector is a disk made from natural diamond (density 3.51 g/cm^3^) with a radius ranging from 1.0 to 2.2 mm and a thickness ranging from 0. 2 to 0.4 mm.^(26)^ ${\text{Li}}_{2}B_{4}O_{7}$ cylindrical pellets (4.6 mm diameter×0.8 mm thickness) are used in radiotherapy dose measurements.^(27)^ In order to quantify the influence of detector thicknesses on the calculated response, we adapted an approach as applied in a previously published work^(3)^ due to limitations associated with the DOSRZnrc user‐code. LiF, ${\text{Li}}_{2}B_{4}O_{7}$, and ${\text{Al}}_{2}O_{3}$ detectors are modeled as cylindrical shells of thickness 1 mm and height 2 mm along the transverse axis of the source. The phantoms considered are water, polystyrene, and plastic water. Absorbed dose and collision kerma to these detectors are calculated at r=1 and 15 cm. The DOSRZnrc‐based collision kerma values are statistically identical to the FLURZnrc‐based collision kerma values. This suggests that detector dimensions do not affect the calculated values. In the case of diamond detector, the calculations are carried out for 0.2 mm and 0.4 mm thicknesses separately (height is 2 mm). DOSRZnrc calculations using these thicknesses show collision kerma values comparable to those obtained using the FLURZnrc user‐code. Whereas the absorbed dose calculated for the 0.2 mm thick diamond detector is smaller by about 1% when compared to the collision kerma. In the case of 0.4 mm thick diamond detector, both collision kerma and absorbed dose are statistically identical.

## IV. CONCLUSIONS

Absorbed‐dose energy dependence of solid‐state detector materials such as diamond, LiF, ${\text{Li}}_{2}B_{4}O_{7}$, and ${\text{Al}}_{2}O_{3}$ for the ${}^{137}\text{Cs}$ RTR brachytherapy source is studied using the Monte Carlo‐based EGSnrc code system. Beam quality correction $k_{QQ_{0}}\;(r)$, which reflects absorbed‐dose energy dependence of the detector, shows a gradual decrease with r for the LiF detector (decrease is about 2% over the distance range of 1‐15 cm). Diamond detector shows a gradual increase in $k_{QQ_{0}}\;(r)$ with r (about 3% larger than unity at 15 cm). For ${\text{Al}}_{2}O_{3}$ detector, $k_{QQ_{0}}\;(r)$ decreases with r steeply (about 14% over the distance range of 1‐15 cm). ${\text{Li}}_{2}B_{4}O_{7}$ does not show energy dependence. The study shows that some solid‐state detectors demonstrate distance‐dependent $k_{phan}\;(r)$ values, but the degree of dependence depends on the type of solid phantom and the detector.

## ACKNOWLEDGMENTS

The authors would like to thank Dr. D. N. Sharma, Director, Health, Safety & Environment Group, Bhabha Atomic Research Centre (BARC), and Mr. D. A. R. Babu, Head, Radiological Physics & Advisory Division, BARC for their encouragement and support throughout the study.
